# The feasibility of non-contrast enhanced plus contrast-enhanced computed tomography in discriminating invasive pure ground-glass opacity from pre-invasive pure ground-glass opacity

**DOI:** 10.1186/s13019-020-01159-2

**Published:** 2020-07-06

**Authors:** Shanshan Li, Jinming Yu, Xue Meng, Lingfei Liu, Liang Xu, Liheng Liu, Haiying Yu, Yongsheng Gao, Zhen Zhang

**Affiliations:** 1 Shandong University, Shandong Cancer Hospital and Institute, 44 Wenhua West Road, Ji’nan, Shandong 250117 People’s Republic of China; 2 Liaocheng Traditional Chinese Medicine Hospital, 1 Culture Road, Dongchangfu district, Liaocheng, Shandong 252000 People’s Republic of China

**Keywords:** Ground-grass opacity (GGO), Adenocarcinoma, Mean computer tomography value, Volumetric CT

## Abstract

**Background:**

Invasive pure ground-glass opacity and pre-invasive pure ground-glass opacity have different 5-year overall survival rate and risk of lymph node metastasis and the extent of resection. It is difficult to discriminate these nodules since they share similar CT features and may occur concurrently. The objectives of this study were to investigate the feasibility of non-contrast enhanced plus contrast-enhanced computed tomography in discriminating invasive pure ground-glass opacity from pre-invasive pure ground-glass opacity.

**Methods:**

We retrospectively examined 90 patients with pure ground-glass opacity who underwent non-contrast enhanced and contrast-enhanced CT according to a simplified protocol (one non-contrast enhanced measurement and two contrast-enhanced measurements at 30 s and 60 s after contrast injection) from 2015 to 2019. All imaging examinations were analyzed using three-dimensional computer-aided volume. Two independent samples t tests, one-way analysis of variance, chi-square test and logistic regression were used for analysis. A receiver operating characteristic curve was used to determine the optimal cut-off value of mean CT attenuation for differentiation of groups and to obtain diagnostic value.

**Results:**

(1) The CT values of one non-contrast-enhanced, two contrast-enhanced and volume measurements between two groups had statistically significant differences (*P* < 0.001). (2) At the 30-s scan, there were more nodules in the pre-invasive group with no enhancement than in the pre-invasive group, which was statistically significant. (3) The CT value of 60-s scan was independent predictor of invasive adenocarcinoma (*P* = 0.019).

**Conclusions:**

Non-contrast enhanced plus two contrast-enhanced CT based on volume measurements can differentiate invasive pGGO from pre-invasive pGGO.

## Background

In 2011, the concepts of adenocarcinoma in situ (AIS), minimally invasive adenocarcinoma (MIA), invasive adenocarcinomas (IA) were introduced in a new classification published by the International Association for the Study of Lung Cancer/American Thoracic Society/European Respiratory Society (IASLC/ATS/ERS) [1]. With advances in imaging techniques, more GGO (especially pure GGOs) are diagnosed. Approximately 60–80% of pure ground-glass nodules (pGGNs) tend to be pre-invasive adenocarcinoma [2],while the rest of pGGNs are pathologically classified as invasive adenocarcinomas [3]. The complete resection of pre-invasive pGGNs results in 100% disease-specific survival [2], in contrast, even after resection of invasive nodules, the 5-year overall survival rate is only 49–84% [4]. Also, the risk of lymph node metastasis and the extent of resection are very different between pre-invasive and invasive adenocarcinoma [5]. Therefore, the preoperative diagnosis between these nodules is extremely important. As a non-invasive examination, contrast-enhanced CT has shown improved accuracy in the diagnosis of solitary pulmonary nodules [3, 6, 7]. The CT value of ground-glass opacities has previously been verified to be a risk factor associated with future changes in the tumor(s) [8]. At present, the number of studies assessing the enhancement characteristics of pGGNs is limited. We retrospectively examined imaging data of patients from 2015 to 2019 who underwent a general clinical protocol of thoracic scanning: one non-contrast enhanced measurement and two contrast-enhanced CT measurements at 30 s and 60 s after contrast injection. We aimed to identify whether this type of contrast-enhanced CT could predict the invasiveness of pGGO.

## Methods

### Study population

This retrospective study was approved by the institutional review board of Shandong Cancer Hospital affiliated to Shandong University, and informed consent was waived. We included 90 patients with pGGO seen in our hospital between October 2015 and October 2019. Patients were aged 26–83 years (median, 55 years), including 55 female patients and 35 male patients, and were diagnosed with certified different pathological types of adenocarcinoma (AIS, MIA, IAC) by resection or biopsy histology, and had standard thorax CT scanning (non-contrast enhanced and two contrast-enhanced CT: scanning at 30-s and at 60-s). The reason for three scans was additional tumors, such as lung cancer, cervical cancer, and breast cancer. The cases of AAH were excluded because of the small number. Refer to previous studies [9], these (AIS and MIA) are referred to as pre-invasive adenocarcinomas and other types of tumors are referred to as invasive adenocarcinomas (IAC).

### CT imaging techniques

Thoracic CT scans were performed using a 128-row CT scanner (MX 8000, Philips Medical Systems) with 1.25 mm collimation, 1.375 pitch, 120-kV tube voltage, 200-mA tube current, 512  × 512 matrix, and 0.5-s scanning time. Axial images of 1.0 mm thickness with 0.5 mm spacing were reconstructed. Three CT scans were performed for each patient: one before the administration of contrast injection, and one after both 30 s and 60 s from the administration of intravenous contrast injection (2.5 mL/s, 1.5 mL/kg). Using an interactive model with the Philips’ volume measurement software (Lung Nodule Assessment), two experienced chest radiologists measured the volume of GGNs. Vessels and bronchioles within the nodule were eliminated manually from the images used. Medical records were reviewed retrospectively to investigate the clinical characteristics, CT values of GGO, histopathological results, and follow-up outcomes.

### Pathological diagnoses

The specimens of GGO nodules were routinely fixed in 10% formalin and processed into paraffin blocks for pathological examination. Two experienced lung pathologists made the pathological diagnoses. All pGGO nodules were diagnosed as AIS, MIA or IAC according to the new IASLC/ATS/ERS classification [1].

### Statistical analysis

All statistical analyses were conducted using SPSS (version 19.0). As AIS and MIA have similar prognoses, these two subtypes of pGGNs were combined into a single group (pre-invasive) and compared with IAC (invasive). All data were recorded as means ± standard deviation. Significant differences were assessed using independent samples t-test and the Chi-square test for continuous variables. A binary logistic regression analyses were used to identify the independent factors for the stratification of invasive and non-invasive adenocarcinoma. Characteristics with *P* values of less than 0.05 on one-way ANOVA were used as the input variables for the multiple logistic regression analysis. Finally, Receiver operating characteristic (ROC) curve analysis was performed to determine the cut-off value, sensitivity, specificity, and accuracy of the significant predictive factors. Statistical significance was set at a *P* value of < 0.05.

## Results

**Fig. 1:**
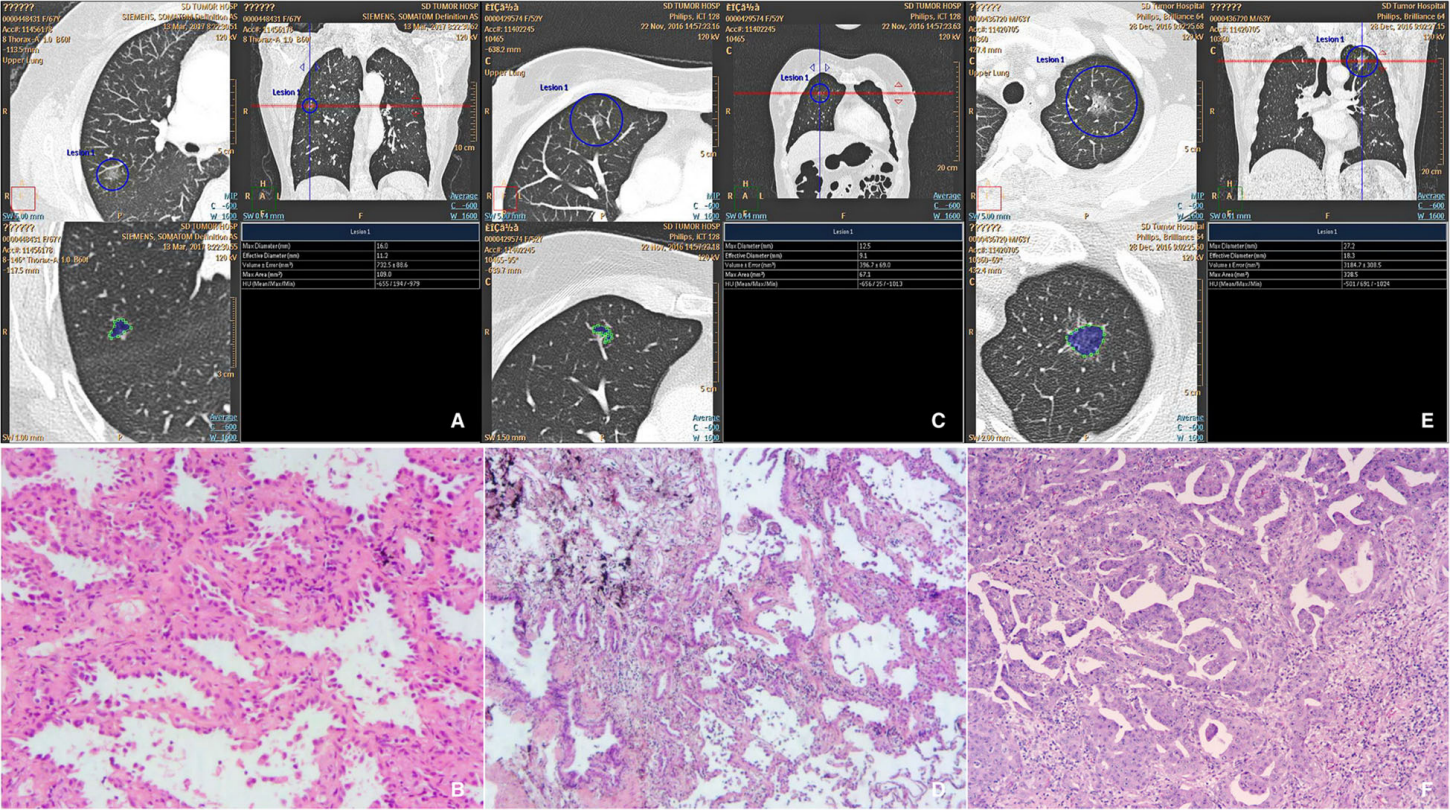
volume-rendering technique CT image and Pathological sections (hematoxylin-eosin staining,original magnification× 40) (**a**, **b**) CT image of pure ground-glass nodules opacity in the right medium lobe of a 67-year-female. Image show that vessels passed through the GGO were eliminated manually. Pathological results confirmed the diagnosis of adenocarcinoma in situ (AIS). **c**, **d** CT image of pure ground-glass nodules opacity in the right medium lobe of a 52-year-female. Pathological results confirmed the diagnosis of minimally invasive adenocarcinoma (MIA). **e**, **f** CT image of pure ground-glass nodules opacity in the left upper lobe of a 63-year-male. Pathological results confirmed the diagnosis of invasive adenocarcinoma (IAC)

**Fig. 2:**
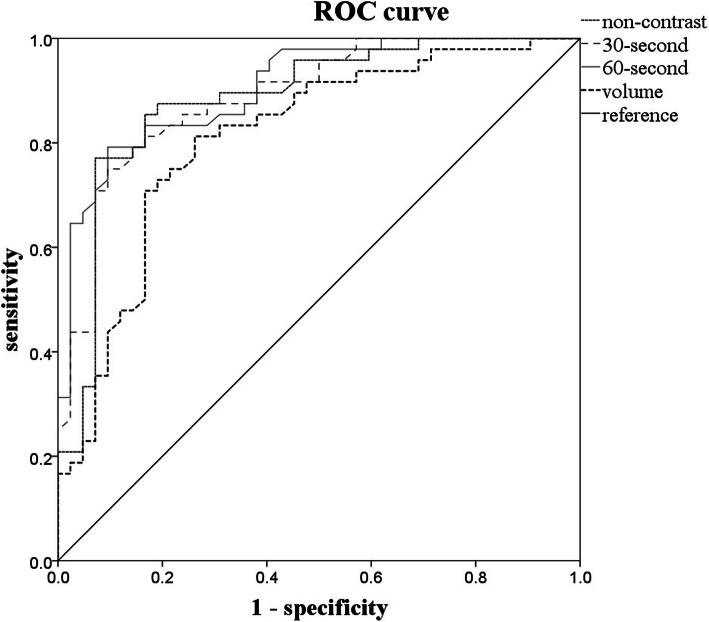
ROC curves of three scans and volumes for indicating invasive adenocarcinoma

The pathological examination observed IAC (Fig. 1) in 48 nodules (53.3%), MIA (Fig. 1) in 22 nodules (24.4%), and AIS (Fig. 1) in 20 nodules (22.3%) (Table 1). The pre-invasive and invasive groups were comprised of 48 and 42 nodules, respectively. The volumes of pre-invasive and invasive pGGO are presented in Table 1. There was significantly difference (*P* < 0.001) between the two groups. The area under the curve (AUC) (Fig. 2) from ROC was 0.811, and the optimal cut-off value of 814.5 mm^3^ for differentiation between pre-invasive and invasive nodules, with a sensitivity of 81.3% and a specificity of 73.8%. In the 30-s scan, 27 out of 42 pre-invasive pGGOs (64.3%) had an increased CT value and 40 out of 48 invasive pGGOs (87.5%) had increased CT value, there was significant difference between the groups (*p* = 0.039). In the 60-s scan, 74 out of 90 of nodules (82.2%) showed increased CT value, excluding nine pre-invasive nodules and seven invasive nodules. The mean CT values of pre-invasive pGGO of one non-contrast enhanced and two contrast-enhanced scans are presented in Table 2. (1) The non-contrast enhanced CT values of pre-invasive and invasive nodules were significantly different (*p* < 0.001). The AUC (Fig. 2) of non-contrast enhanced scans from ROC was 0.884, and the cut-off values of − 605HU were optimal for differentiation between pre-invasive and invasive nodules, with a sensitivity of 85.4% and a specificity of 83.3%. (2) The contrast-enhanced CT values between groups were significantly different (*p* < 0.001) (Table 2). The results of the ROC analysis are shown in Table 3. The AUC of 30-s scan was 0.888 and the cut-off value was − 563.0 HU, with a sensitivity of 75% and a specificity of 90.5%. The AUC of 60-s scan was 0.906 and the cut-off value was − 575.5 HU, with a sensitivity of 79.2% and a specificity of 90.5%.According to one-way ANOVA, the mean CT values of one non-contrast enhanced, two contrast-enhanced and volume measurements had statistically significant differences (*P* < 0.001). Multivariate logistic regression analysis revealed the mean CT value of 60-s scan was independent predictor of invasive adenocarcinoma (*P* = 0.019).

**Table 1:** characteristics of patients with pre-invasive (AIS,MIA) and invasive adenocarcinoma(IAC)

	total	preinvasive	invasive
Sex			
male	35	16	19
female	55	26	29
Median age	55.83 ± 9.99	56.29 ± 10.07	55.44 ± 9.39
Type of pathology			
AIS	20	20	
MIA	22	22	
IAC	48		48
Volume		0.85 ± 1.00	2.42 ± 2.11

**Table 2:** the CT values of pGGO

	non-contrast	30s’scan	60s’scan
Pre-invasive	− 654.79 ± 68.85	− 638.76 ± 67.97	− 640.98 ± 62.17
invasive	− 538.23 ± 70.70	− 510.46 ± 78.33	− 502.23 ± 82.41
p	<0.001	<0.001	<0.001

**Table 3:** ROC curves of three scans and volume

	AUC	sensitivity	specificity	cut-off value
plain	0.884	85.4%	83.3%	−605
30s	0.888	75%	90.5%	−563
60s	0.906	79.2%	90.5%	−575.5
volume	0.811	81.3%	73.8%	814.5

Figure 1, Figure 2.

## Discussion

The growth and metastasis of tumors depends on angiogenesis. Measuring the enhancement degree of pulmonary tumors has proven to be helpful in distinguishing pulmonary nodules in contrast-enhanced CT, due to distinct differences in vascularity and vasculature [10]. In past research, dynamic enhancement CT scans were often used to evaluate lung nodule enhancement patterns. They currently are not commonly used because of the high radiation exposure, even though they can contribute to a better understanding of the enhancement features of lung nodules. The classic enhancement CT scans (30 s, 60 s) can reflect the changes of blood supply of lung nodule to some extent. In our study, we retrospectively analyzed non- and contrast-enhanced CT (30-s scans and 60-s scans) values of pGGOs that manifested this pattern are able to distinguish invasive from pre-invasive pGGOs.

Some studies have reported that 3D volume measurements can evaluate tumors better than the conventional 2D diameter measurements in pulmonary nodules, with good inter-observer reproducibility [11–13]. The cellularity of pure GGO is too low to evaluate and is usually accompanied by blood vessels and bronchioles. In this study, we chose a thin-section and automated volumetric measurement of GGO to evaluate various radiologic parameters [14]. Then we manually eliminated pulmonary vessels and bronchioles in ROIs from the images to perform objective assessments. Our study revealed that the difference of the volumes between pre-invasive and invasive pGGOs is significantly (p <0.001). The area under the curve (AUC) from ROC was 0.811, and the cut-off value was 814.5 mm^3^ with a sensitivity of 81.3% and a specificity of 73.8%. The study of Han L et al. [15] showed that the area under the curve (AUC) from ROC was 0.781 and 1.349 mm^3^ was the optimal cut-off value for volume of the nodule to differentiate MIA from IAC. According multivariate logistic regression analysis, the volume was not independent predictor of invasive adenocarcinoma, which was consistent with the conclusion by Ji Ye Son et al. using virtual non-contrast imaging of dual-energy CT [16].

In our study, the non-contrast enhanced CT value of invasive pGGO was significantly greater than that of pre-invasive lesions. This finding is also consistent with several of other previous studies [11, 17]. The attenuation of the GGO nodule in CT is a combination of the attenuation of soft tissue and air. Usually, AIS and MIA has more air space and fewer cellular components, thus they usually have significantly lower CT attenuation than IAC. We concluded that − 605 HU was the optimal cut-off values for differentiation between pre-invasive and invasive, with a sensitivity of 85.4% and a specificity of 83.3%. Kitami A, et al. [18] reported that the mean-CT value of GGO was a significant predictive factor for invasiveness of the tumor. A mean CT value of > − 600 HU was highly indicative of an invasive lesion.

The findings of the 30-s scans in our study were not consistent with former reports. We found that nearly half of the pre-invasive pGGOs (15/42) had no enhancement, and that there were only 8(8/48) IAC which had no enhancement. The difference between pre-invasive and invasive pGGOs was significant (*p* <0.001). In cases with enhancement, the CT attenuation between pre-invasion and invasion was significantly different. Yamashita et al. indicated that the maximum enhancement CT value of lung carcinomas was mainly correlated to the number of small vessels, with diameters of 0.02–0.10 mm [19]. These newly formed tumor blood vessels may have only a single layer of endothelial cells, which are relatively immature and frequently demonstrate fissures as well as a lack of basilar membrane [20]. Compared with AIS and MIA, which are defined as a small adenocarcinoma ≤5 mm in diameter, IACs had more tumor components, which means more vascularity and greater permeability, thus leading to earlier and greater enhancement.

Our study revealed that there was a significant difference between pre-invasive and invasive tumors using the 60-s scans, which is consistent with Feng Gao’s report [21]. He used the same scan-time (60s) and indicated no statistical difference between the presurgical diagnosis based on contrast-enhanced CT images and the pathological diagnosis. In the end, the AUC values in our study obtained from ROC for three scans were 0.884, 0.888 and 0.906 (Fig. 2), respectively. The AUC value of the 30-s or 60-s scan was greater than the non-contrast enhanced scans. The AUC of the 60-s scan was the greatest. Furthermore, logistic regression analysis showed that the CT value of the 60-s scan could be the most significant predicting factor. Ying Zhang et al. using 85-s scan and higher energy (140 keV) of DECT revealed that enhanced monochromatic CT number was a significant factor indicating IAC, but it had lower AUC and specificity (AUC =0.635, sensitivity =46.7%, specificity =87.5%, threshold = − 476.4 HU) [22].

The limitation of our study was that it was a retrospective study with a relatively small sample size. Future studies with a substantially larger number of patients are necessary. In addition, predominant lepidic histology is being actively studied as a unique group because its behavior is less aggressive than other subtypes of adenocarcinomas [5, 23–26]. This subtype of adenocarcinoma typically presents with ground glass opacity (GGO) [23, 24]. Due to the limited number of cases enrolled in our study, this group could not be studied separately. Such a trial is being planned in our next research.

## Conclusions

Non-contrast enhanced plus contrast-enhanced computed tomography with CT value and volume of nodule measurement can predict pathological invasiveness and improve understanding and choosing appropriate treatment strategies for the patients with pGGOs.

## Data Availability

This retrospective study used data from the Shandong Cancer Hospital.

## Abbreviations

CT: Computed tomography
GGO: Ground-grass opacity
pGGO: pure Ground-grass opacity
mGGO: Mixed Ground-grass opacity
AIS: Adenocarcinoma in situ
MIA: Minimally invasive adenocarcinoma
IAC: Invasive adenocarcinomas
IASLC/ATS/ERS: The International Association for the Study of Lung Cancer/American Thoracic Society/European Respiratory Society
AHH: Atypical adenomatous hyperplasias
ROC: Receiver operating characteristic
AUC: The area under the curve
